# Effect of MA01 rhamnolipid on cell viability and expression of quorum-sensing (QS) genes involved in biofilm formation by methicillin-resistant *Staphylococcus aureus*

**DOI:** 10.1038/s41598-022-19103-w

**Published:** 2022-09-01

**Authors:** Fatemeh Saadati, Shahab Shahryari, Naeema Mohseni Sani, Davoud Farajzadeh, Hossein Shahbani Zahiri, Hojatollah Vali, Kambiz Akbari Noghabi

**Affiliations:** 1 National Institute of Genetic Engineering and Biotechnology (NIGEB), P. O. Box 14155-6343, Tehran, Iran; 2 Department of Cellular and Molecular Biology, Faculty of Biological Sciences, Azarbaijan Shahid Madani University, Tabriz, Iran; 3 Department of Anatomy & Cell Biology and Facility for Electron Microscopy Research, McGill University, 3640 Street, Montreal, QC H3A 0C7 Canada

**Keywords:** Microbiology, Pathogenesis

## Abstract

A group of biosurfactants, called rhamnolipids, have been shown to have antibacterial and antibiofilm activity against multidrug-resistant bacteria. Here, we examined the effect of rhamnolipid biosurfactants extracted from *Pseudomonas aeruginosa* MA01 on cell growth/viability, biofilm formation, and membrane permeability of methicillin-resistant *Staphylococcus aureus* (MRSA) ATCC6538 bacterial cells. The results obtained from flow cytometry analysis showed that by increasing the concentration of rhamnolipid from 30 to 120 mg/mL, the cell viability decreased by about 70%, and the cell membrane permeability increased by approximately 20%. In fact, increasing rhamnolipid concentration was directly related to cell membrane permeability and inversely related to cell survival. Microtiter plate biofilm assay and laser scanning confocal microscopy analysis revealed that rhamnolipid, at a concentration of 60 mg/mL, exerts a reducing effect on the biofilm formation of Staphylococcus aureus. Real-time PCR analysis for monitoring the relative changes in the expression of agrA, agrC, icaA, and icaD genes involved in biofilm formation and related to the quorum-sensing pathway after treatment with rhamnolipid indicated a reduced expression level of these genes, as well as sortase A gene. The results of the present study deepen our knowledge regarding the use of microbial natural products as promising candidates for therapeutic applications.

## Introduction

In recent years, bacteria such as *Pseudomonas aeruginosa*, *Staphylococcus aureus*, and *Escherichia coli* have been among the most important causes of nosocomial infections, showing the most resistance to antibiotics and mortality of hospitalized patients^1–4^. Methicillin-resistant *Staphylococcus aureus* (MRSA) bacteria are one of the most prominent biofilm-forming pathogens^5^. When bacteria form biofilms, the bacteria in the biofilm status show a higher resistance to antibiotics than the free-floating plankton form. For this reason, the eradication of these kinds of bacteria is laborious and of great therapeutic importance^1^. MRSA is a methicillin-resistant strain and one of the most pathogenic strains of antibiotic-resistant *Staphylococcus aureus*. This bacterium is also able to form biofilms in addition to its resistance to the antibiotic methicillin. Hence, its pathogenic effects are intensified after biofilm formation^6^. In fact, biofilm-producing bacteria are a source of persistent or chronic infections^7^. Microorganisms typically use cellular interactions to adapt to environmental changes, and cell-to-cell communication also plays a significant role in biofilm formation. Some environmental factors such as nutrient availability, temperature, osmolarity, iron ion concentration, light, and acidity can also affect biofilm formation. Indeed, environmental parameters cause the bacteria to form a biofilm to protect themselves against antibiotics and the immune system. Biofilm formation requires altering the expression of a set of genes in a bacterium^8–10^. The cells in the biofilm can escape the immune system and become more resistant to antimicrobial compounds. For this reason, the study of bacterial biofilms and potential inhibitors of biofilm formation is of great importance for therapeutic purposes. Various systems and an organized framework regulate the genes involved in biofilm formation. One of these is the quorum-sensing signaling system. Quorum sensing is a cell-to-cell communication system under which bacteria form aggregates and then exhibit specific behaviors and functions against the external environment by specific signal molecules called inducers^11–13^. The quorum mechanism allows bacteria to perceive the density of the surrounding bacterial community and respond to them by arranging different genes in a coordinated manner. Several genes are involved in biofilms formation in coordination with each other. The main adhesion-associated genes (microbial surface components that recognize adhesive matrix molecules) involved in cell aggregation in the biofilm are eno, fnbB, icaAD, fnbA, fib, ebpS, clfB, bbp, bap, clfA, cna, pls, sasC, sasG, icaBC^14^. One of the trails involved in the biofilm-formation by *S. aureus* is the icaABCD operon, and the icaA, icaB, icaC, and icaD genes are prominently involved in the biofilm formation of this bacterium^15–17^. The icaD gene is located between the icaA and icaB genes. The icaA gene has a transcriptional role, but its transcriptional activity is low, and the icaD gene helps increase its activity for slime generation^18^. The ica operon encodes proteins and enzymes responsible for intercellular adhesion genes and biofilm production. While the transcribed proteins IcaA, IcaD, IcaB, and IcaC have separate functions, they are correlated in the synthesis of intercellular adhesive polysaccharides^19^.

The agr system plays a significant role in pathogenesis by regulating virulence factors, biofilm formation, and the resistance of MRSA^20^. Sortase A is another crucial enzyme present at the cell surfaces of gram-positive bacteria such as *S. aureus*, which plays a substantial role in pathogenesis and biofilm formation^21^.

Rhamnolipids (RLs), as glycolipid-type biosurfactants, are mainly produced by different *P. aeruginosa* strains. These kinds of biosurfactants have antiviral, anti-biofilm, hemolytic, antimicrobial, anti-adhesive, and antifungal properties^22–24^. It has been shown that they interact with the non-polar portion of the *S. aureus* cell membrane. Indeed, rhamnolipids insert their shorter acyl tails into the cell membrane, distorting its configuration^25^. Studies have shown the dramatic effect of rhamnolipid biosurfactants on the inhibition of biofilm formation in *S. aureus*. It has also been reported this antibacterial agent can disperse the biofilm architecture of various bacteria such as *Bordetella bronchiseptica*, *Bacillus pumilus*, *S. aureus*, *Listeria monocytogenes*, and *Salmonella enteritidis*^26–28^. In the present study, the effect of rhamnolipid biosurfactant, produced by *P. aeruginosa*, on inhibiting the growth and *S. aureus* biofilms formation was studied. The changes occurred in the expression of genes involved in the quorum sensing pathway affecting biofilm formation and cell membrane permeability was examined before and after treatment with rhamnolipid biosurfactant.

## Methods

### Bacterial cultivation

Initially, the stock of *P. aeruginosa* MA01, stored in the freezer at − 70 °C, was cultured on a nutrient agar medium (Merck Co.) and incubated overnight at 37 °C. The bacteria were then inoculated into a 100 mL Erlenmeyer flask containing approximately 20 mL of sterile liquid nutrient broth medium. Afterward, it was placed in a shaking incubator at 37 °C and 180 rpm overnight.

### Rhamnolipid production and extraction

To produce rhamnolipid biosurfactant, a salt-based medium was used as contained (g/L): NANO_3_ 3; yeast extract 1; KH_2_PO_4_ 0.25; MgSO_4_·7H_2_O 0.25; and soybean oil (carbon source) 40. Crude biosurfactant was achieved using acidic precipitation and solvent extraction method^29^. After three days of incubation at 30 °C and 180 rpm, cells were removed from the culture broth by centrifugation at 10,000×*g* for 15 min at 4 °C. The cell-free supernatant was acidified with 6 N HCl to pH 2 and kept overnight at 4 °C to increase biosurfactant precipitation. The obtained precipitate was separated by centrifugation (18,000×*g*, 30 min, 4 °C) and extracted several times with ethyl acetate at room temperature. The solvent was completely vaporized in a vacuum. The crude biosurfactant was collected as a viscous brown substance and weighted as g/L.

### Antibacterial assay

#### Pre-cultivation of MRSA

Initially, linear cultures of the test bacterium were given on LB agar medium from the MRSA stock stored at − 80 °C and then incubated at 37 °C overnight. After bacterial growth, single colonies were removed and inoculated into a 50 mL Falcon containing 10 mL of liquid LB medium and incubated at 37 °C at 180 rpm overnight.

#### Determination of minimum inhibitory concentration (MIC)

First, a rhamnolipid concentration of 12% (120 mg/mL of filtered rhamnolipid) (Merck Millipore Millex Syringe filter, pore size 0.22 µm) was prepared in the TSB medium containing 0.2% glucose, and then different dilutions up to 0.01% were made. 16-h pre-cultures of MRSA bacteria were equivalent to 0.5 McFarland standard used and added to each dilution. After 15 h of incubation at 37 °C at 180 rpm, each dilution was diluted by adding PBS 50 mM, pH 7.4, and a standard plate count was performed to estimate the population density of bacteria. Bacterial colonies were carefully counted on each plate, and the results were calculated according to the following formula.

$${\text{cfu/mL}} = ({\text{no.}} \, {\text{ of}} \, {\text{colonies}} \times {\text{dilution}} \, {\text{factor}})/{\text{volume}} \, {\text{of}} \, {\text{culture}} \, {\text{plate}}$$

### Biofilm assay

#### Microtiter plate assay for assessment of MRSA biofilm formation

The microtiter plate phenotypic method was used to determine the biofilm formation of MRSA bacteria before and after treatment with a rhamnolipid biosurfactant. For this purpose, 96-well microtiter plates were used. One hundred microliters of dilutions, according to the dilutions obtained from MIC, namely 24%, 12%, 6%, and 3%, were poured into the wells. Then, 2 µL of a 10% glucose solution was added to each well, and MRSA (equivalent to half McFarland standard) was inoculated into each well. After 48 h, the formed biofilms were stained with crystal violet. To this end, after discharging the planktonic cells and washing twice with sterile physiological saline, 200 µL of 96% ethanol were added to each well. After 20 min and draining the ethanol, the plate was allowed to dry at laboratory temperature. After drying the plate, 100 µL of 0.2% crystal violet were added to the wells for staining and emptied after 15 min. Washing with sterile deionized water was performed twice, and then 200 µL of 33% acetic acid were added to each well to dissolve the formed biofilms. The plate was read on a multi-well scanning spectrophotometer (ELISA reader) at 600 nm.

#### Confocal laser scanning microscopy (CLSM)

Confocal laser scanning microscopy is a valuable tool for studying bacterial biofilms, allowing real-time visualization of living specimens^30^. 24-well plates with coverslips were used and poured as follows:

(1) For control: 450 µL of TSB medium, 0.2% glucose, and 1.5 × 10^8^ colony forming units (CFU/mL) of a 16 h MRSA culture suspension (equivalent to 0.5 McFarland turbidity standard), and (2) For treatment, 450 μL of 24% and 6% rhamnolipid concentrations dissolved in TSB containing 0.2% glucose was poured into a well and inoculated with 1.5 × 10^8^ CFU/mL of the bacterial suspension. The microtiter plates were then incubated at 37 °C for 48 h. After 48 h, planktonic cells were emptied and washed twice with PBS. Then, 450 µL of PBS was poured into the wells, and fluorescein diacetate (FDA) (with a concentration of 500 µL per milliliter) was added to plates at 100 μL/well. The plate was wrapped in foil for 20 min and incubated at room temperature. Subsequently, 1 μL of 1 × propidium iodide (PI) was added and incubated for 2 min. Then, the contents of the wells were drained, and after twice washing the coverslips with PBS (50 mM, pH 7.4), the biofilms were prepared for imaging with a confocal microscope.

#### MRSA cell's membrane permeability and viability treated with rhamnolipid

Four different concentrations of 24%, 12%, 6%, and 3% rhamnolipid were prepared with a TSB medium containing 0.2% glucose, and MRSA bacterial suspension (equivalent to 0.5 McFarland turbidity standards) were added to each dilution. The cells were centrifuged at 4000 rpm for 4 min at 4 °C after 15 h of incubation at 37 °C at 180 rpm. The collected cells were washed twice with PBS (50 mM, pH 7.4), and 35 μL of FDA (6 mM) was added to the suspended cells in PBS and incubated for 25 min at room temperature in the dark. Afterward, 6 µL of PI (50X) were added and incubated at room temperature and dark condition for 5 min and read by PAS III flow cytometer. Cell-free PBS was utilized to reset the device. FlowJo software (Tree star, Inc. Ashland, Oregon, USA) was used to analyze the obtained data.

#### Effect of rhamnolipid on expression of genes involved in MRSA biofilm formation

To extract the RNA from MRSA biofilms, the bacterium was first pre-cultivated in liquid LB medium. As a control, only a liquid LB medium was poured into the plates, and rhamnolipid biosurfactant, with a concentration of 3% and 6%, was prepared with LB liquid medium for the treatment. Then the target bacterium (equivalent to 0.5 McFarland turbidity standards) was inoculated into the plates. The plates were incubated at 37 °C for 48 h. The planktonic cells of MRSA were completely removed after 48 h, and 1 mL of PBS was added, pipetting well to remove the biofilm formed at the bottom of the plates. Dissolved biofilms were centrifuged in PBS at 5000 rpm for 20 min. The supernatant was drained, and the cells were suspended in PBS (50 mM, pH 7.4). The lysozyme (50 mg/mL) was then added to them and incubated at 4 °C for 4 min. RNX-PLUS kit (Sinagen Co.) was used to continue the extraction. After RNA extraction from the biofilm, the concentration of RNA extracted from the control and treated cells was read with a Thermo Scientific NanoDrop spectrophotometer. The horizontal gel electrophoresis was performed for further confirmation. BioFact kit (Daejeon, South Korea) was used to synthesize cDNA from extracted RNAs. Then, the expression of genes related to *S. aureus* biofilms (icaA, icaD, agrA, agrC, srtA) was analyzed using qRT-PCR. The sequences and primers used in this study have shown in Table 1.

**Table 1:** Primer sequences used for real-time PCR.

Gene	Primer	Sequence (5′ → 3′)	Product size (this study)
icaD	Forward primer	ATGGTCAAGCCCAGACAGAG	198 bp
icaD	Reverse primer	GCAACACGTATTGTATTGATACTTTCGTCATG	
agrA	Forward primer	TGCGAAGACGATCCAAAACAAAGAG	212 bp
agrA	Reverse primer	CGGATTTCACTGCCTAATTTGATACC	
srtA	Forward primer	GGACAAATCGATTAATGACAATCGCTG	188 bp
srtA	Reverse primer	CGGAATTTGAGGTTTAGCTTGCTG	
agrC	Forward primer	TTGAAGCTATCAACAACGAAATGCG	218 bp
agrC	Reverse primer	CGCAGTAATTAAGCCTTTAATTTCACGT	
V6	Forward primer	TCGATGCAACGCGAAGAA	125 bp
V6	Reverse primer	ACATTTCACAACACGAGCTGACGA	
icaA	Forward primer	TTGCCCACCTTGTGCCCACC	179 bp
icaA	Reverse primer	TGAGGCTGTAGGGCGTTGGGA	

## Results

### Cell growth and rhamnolipid production and extraction

**Figure 1:**
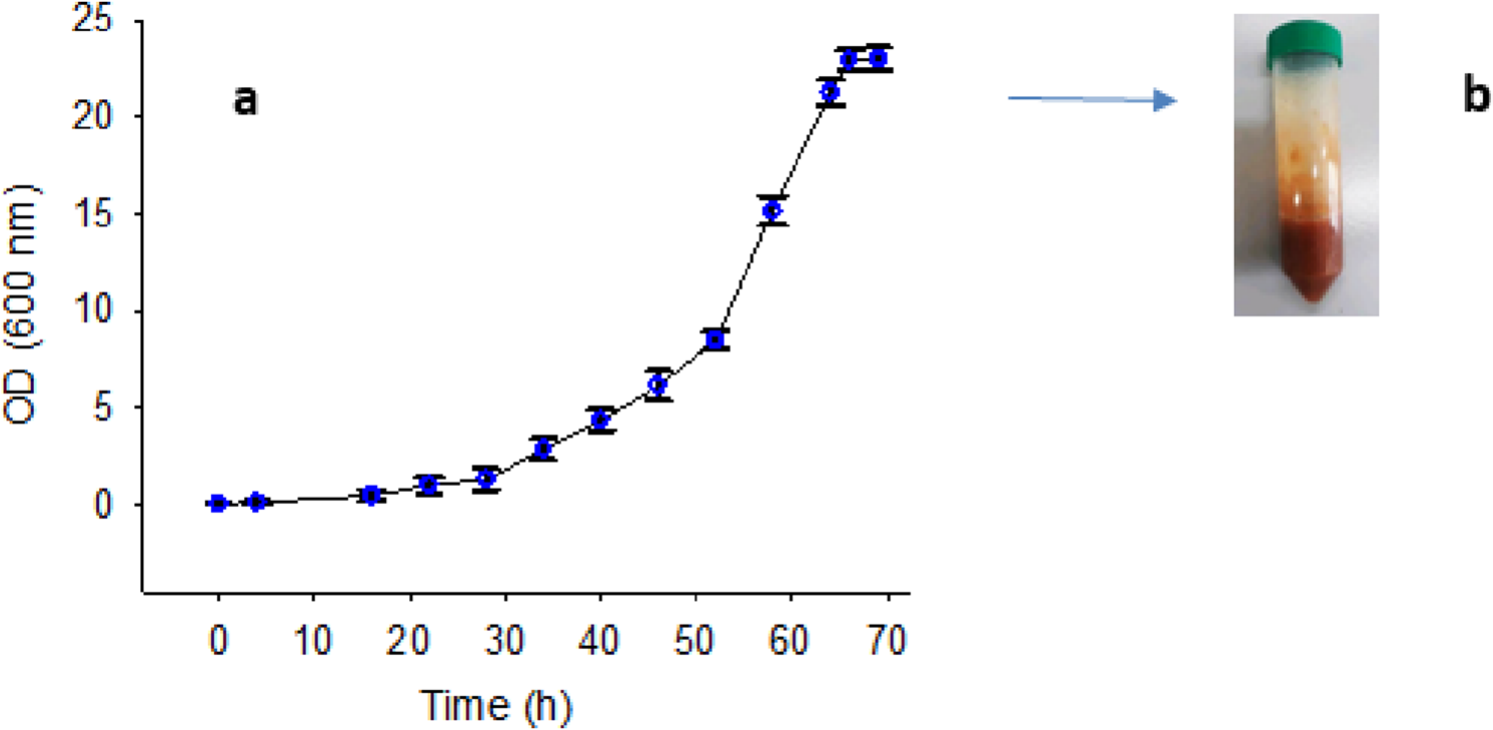
(**a**) *Pseudomonas aeruginosa* MA01 growth curve. Biomass harvesting was performed for rhamnolipid extraction after 70 h, when the bacterium reached stagnant growth. (**b**) Extracted rhamnolipid.

The extraction of rhamnolipid biosurfactant was performed after three days of incubation when the growth of *Pseudomonas* bacteria reached its stationary phase (Fig. 1). After 70 h of inoculation of bacteria with a concentration of 2% into the saline-based medium, a color change (pale green to milky state) was observed in the medium, indicating rhamnolipid production. The resulting change represented that the production of rhamnolipid by the bacterium increased with increasing time, and this process continued until the bacterium reached its steady growth. Based on the results, the overall rhamnolipid production yield was 4 g/L (Fig. 1a,b).

### Determination of the minimum inhibitory concentration (MIC)

**Figure 2:**
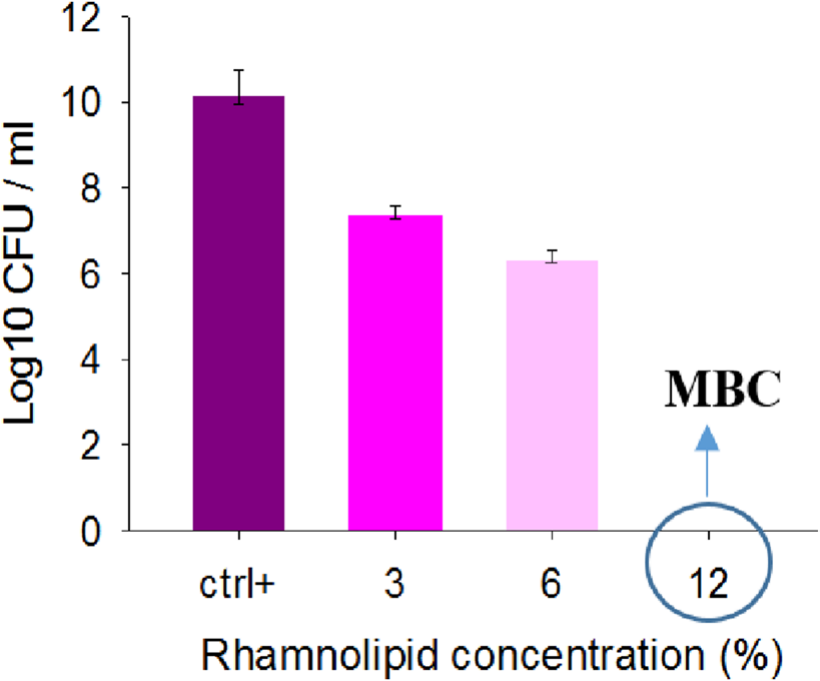
Minimum inhibitory concentration and minimum bactericidal concentration (MBC) of rhamnolipid on MRSA in TSB + glucose medium. A view of control (medium with no rhamnolipid) and bacteria treated with different concentrations of rhamnolipid (3%, 6% and 12%) is shown.

**Figure 3:**
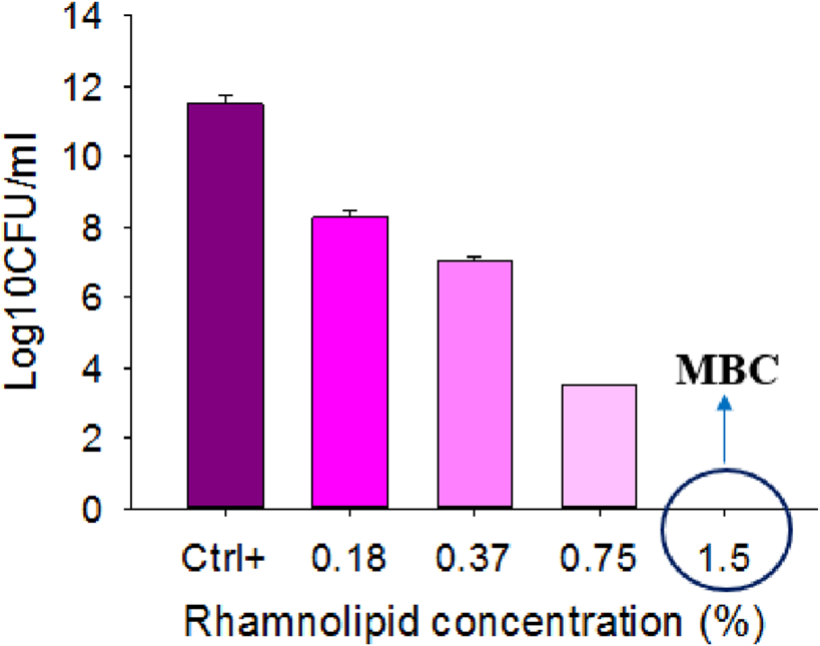
Minimum inhibitory concentration and minimum bactericidal concentration (MBC) of rhamnolipid on MRSA in muller hinton medium. A view of control (medium with no rhamnolipid) and bacteria treated with different concentrations of rhamnolipid (0.18%, 0.37%, 0.75% and 1.5%) is shown.

In order to determine the MIC, the conventional viable cell count or standard plate count method was performed, as rhamnolipid has its color, and the spectrophotometric analysis depends on the opacity, no reliable result is expected to obtain. Figure 2 shows the effect of rhamnolipid at different concentrations on MRSA bacteria. The results showed that with increasing the concentration of rhamnolipid, the growth of MRSA bacteria was further inhibited, and in fact, increasing rhamnolipid concentration was directly related to the growth inhibition of MRSA. Rhamnolipid with a concentration of 3% was selected as the MIC (or minimum inhibitory concentration), and a concentration of 12% was selected as the MBC (or minimum bactericidal concentration). At concentrations lower than 3% rhamnolipid, no inhibitory effect was observed in this study. The results obtained from plate count agar experiments by treating MRSA bacteria with rhamnolipid in Mueller Hinton medium have been shown in Fig. 3. Rhamnolipid with a concentration of 1.5% could kill bacteria, as determined to be MBC concentration. The rhamnolipid concentration of 0.18% was determined as the MIC concentration. The results showed that rhamnolipid in Mueller Hinton medium can have a higher effect on inhibiting the growth of MRSA bacterium. In the TSB + glucose medium, MIC and MBC were equal to 3% and 12%, respectively, while in the MHB medium, MIC and MBC were determined to be 0.18% and 1.5%, respectively (Fig. 3). Therefore, the effect of rhamnolipid depends on the growth medium, as demonstrated more effective performance in the Muller Hinton medium compared to the TSB + glucose medium, inhibiting MRSA bacteria at lower concentrations.

### Analysis of biofilm formation

#### Crystal violet staining

**Figure 4:**
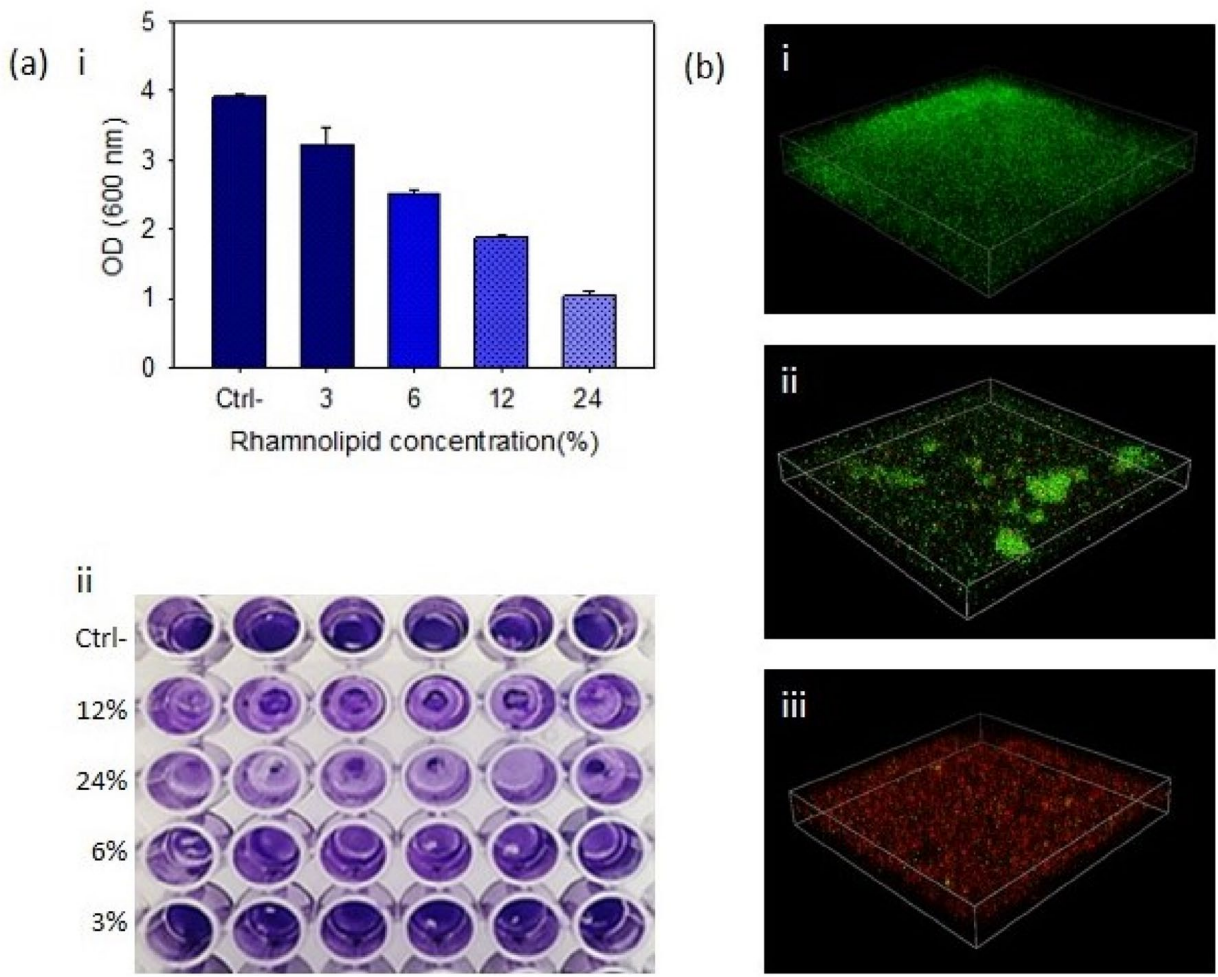
(**a**) Investigating biofilm formation by crystal violet staining. (**i**) Relationship between rhamnolipid concentration and inhibition of MRSA biofilm. (**ii**) Image of biofilm formed by MRSA bacteria in untreated state and treated with different concentrations of rhamnolipid (3%, 6%, 12%, 24%) with a pH of 7. (**b**) Confocal microscope analysis of MRSA biofilms. (**i**) Untreated biofilms, and (**ii**, **iii**) treated biofilm with concentrations of 6% and 24%, respectively.

Staining the biofilm by violet crystal and reading of ODs by ELISA reader at 600 nm showed the biofilm inhibition at different rhamnolipid concentrations. In fact, with increasing rhamnolipid concentrations, biofilm was more inhibited. It was observed that biofilm inhibition was directly related to rhamnolipid concentration (Fig. 4). The results also showed that rhamnolipid with a concentration of 12% inhibited about 50% of the biofilm, and with increasing rhamnolipid concentration a significant increase in biofilm inhibition occurred.

#### Confocal laser scanning microscopy (CLSM) analysis of MRSA biofilms

**Figure 5:**
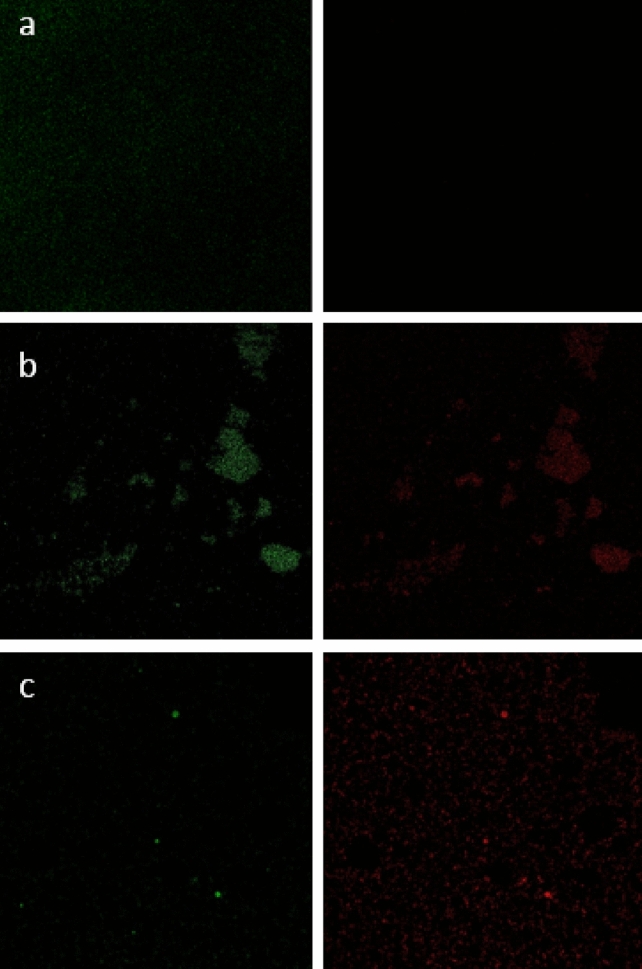
Confocal microscopy two-dimensional images of MRSA biofilms. (**a**) Untreated cells, (**b**) Treated biofilm with concentrations of 6%. (**c**) Treated biofilm with concentrations of 24%.

The confocal microscopy analysis (Figs. 4 and 5) showed that the biofilm formed in treatments with the rhamnolipid concentration of 6% had a significant reduction compared to the control mode, which was consistent with the results of staining with crystal violet. On the other hand, when the biofilm was treated with 24% rhamnolipid, PI penetrated more into the biofilm cells, which means that with increasing concentration, rhamnolipid was able to affect the survival and integrity of the cell membrane.

#### Investigation of cell membrane permeability and viability

**Figure 6:**
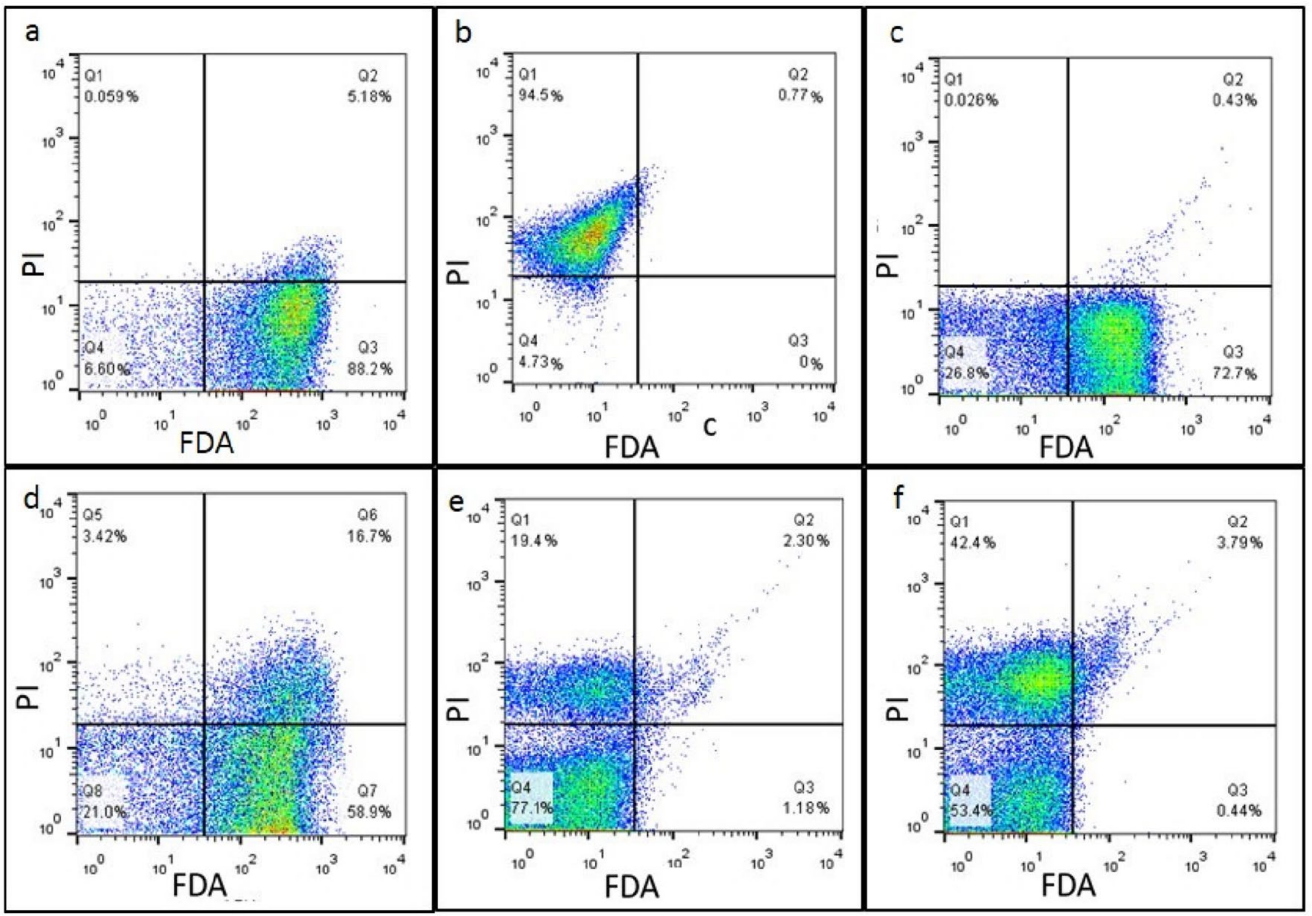
Assessment of the effect of rhamnolipid on viability and permeability of MRSA cell membrane using flow cytometry. (**a**) Live cells, (**b**) dead cells, (**c**) cells after treatment with 3% rhamnolipid, (**d**) cells after treatment with 6% rhamnolipid, (**e**) cells after treatment with 12% rhamnolipid, (**f**) cells after treatment with 24% Rhamnolipid.

PI and FDA dyes were used for fluorescent cell staining and the evaluation of bacterial cell membrane permeability and viability, respectively. MRSA bacterial cells not treated with rhamnolipid were considered as control. The flow cytometry analysis (Fig. 6) showed that increased permeability is directly related to increasing rhamnolipid concentration, and increasing rhamnolipid concentrations is inversely related to bacterial survival. An increase in rhamnolipid concentration could damage the cell membrane integrity. When the rhamnolipid concentration increases, at the same time, cell survival decreases, and permeability increases. Thus, by increasing the concentration of rhamnolipid, the amount of light emitted by the FDA decreases, and due to the damage to the membrane, the PI dye will be able to penetrate the membrane.

#### Real time PCR (qRT-PCR)

**Figure 7:**
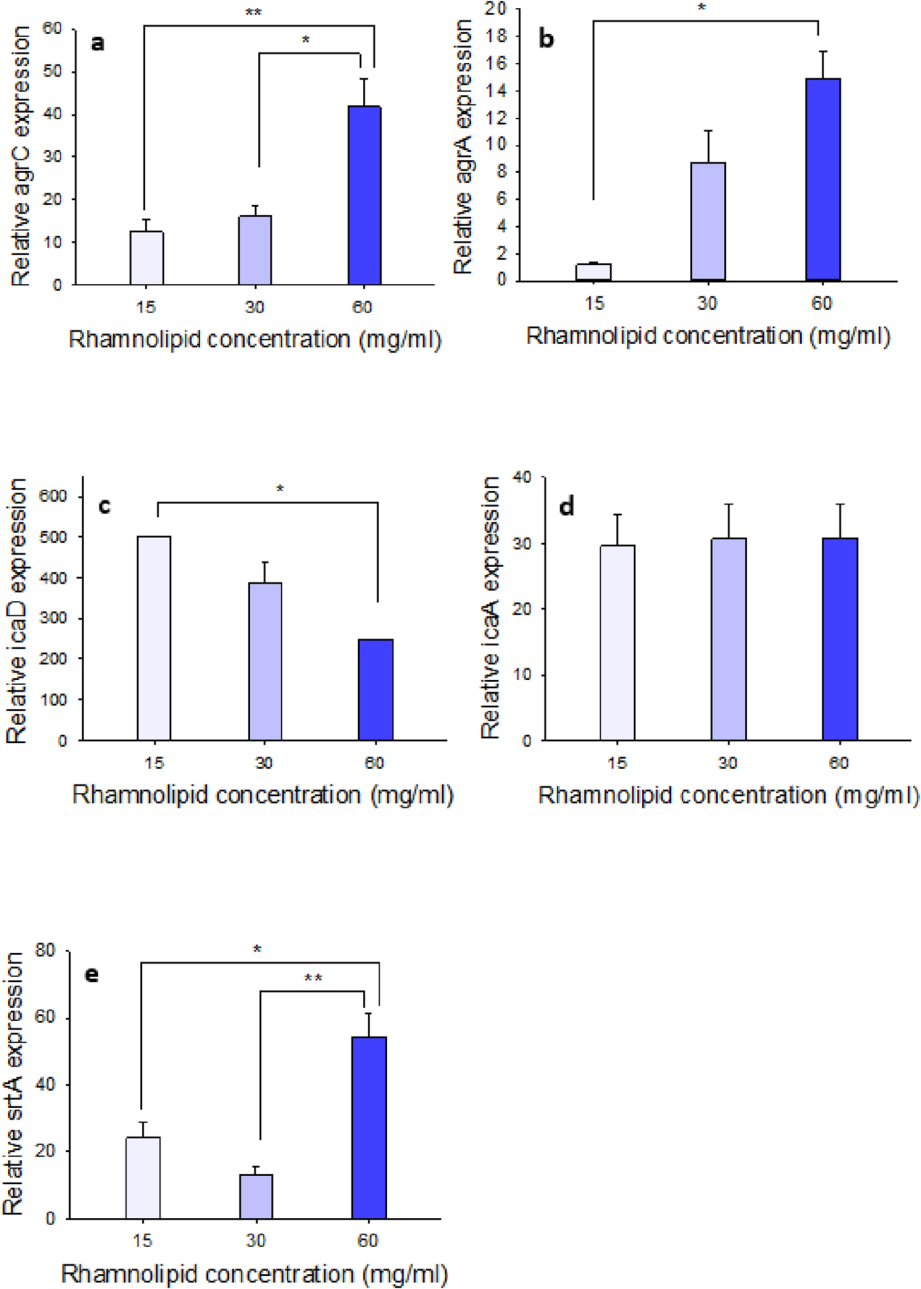
Assessment of the expression of genes involved in biofilm formation of MRSA after treatment with different concentrations of rhamnolipid (15, 30, 60 mg/mL) by using real-time PCR technique; the expression of all studied genes such as; (**a**) agrA, (**b**) agrC, (**c**) icaA, (**d**) icaD, and (**e**) srtA compared with their untreated conditions (with neutral pH) had a significant decrease. There is a statistically significant difference between groups that showed by the symbol *P < 0.05 and **P < 0.005 via one-way ANOVA (Tukey test) and Changes in expressing all columns of each diagram are drawn compared to control mode.

The results of real-time PCR in icaA, icaD, agrA, agrC, srtA genes are shown in Fig. 7. The agr pathway genes, namely agrA and agrC, have been less inhibited by a decrease in rhamnolipid concentration, and in fact, an increase in concentration is directly related to an increase in inhibition. In the ica pathway, no significant difference was observed in the inhibition of the icaA gene between different concentrations. In fact, even concentrations below the MIC of rhamnolipid, 15 mg/mL, were able to inhibit this gene to a large extent, approximately 29-fold. Inhibition of the icaD gene was not dose-dependent, but at lower concentrations of rhamnolipid, a better effect was observed on this gene. As mentioned, icaD helps icaA to make N-acetyl glucose amine, thus forming a biofilm. The results showed that rhamnolipid at different concentrations significantly reduced icaA gene expression, to the extent that a concentration of 15 mg of rhamnolipid reduces the expression of the icaD and icaA genes by 500 times and nearly 30 times, respectively. It can be inferred that biofilm inhibition requires icaD to be inhibited to a greater extent, and therefore after cell treatment with rhamnolipid, this gene cannot help the icaA gene due to reduced expression and biofilm formation is inhibited. Inhibition of the sortase A gene (srtA) was not dose-dependent but was significantly inhibited at low concentrations of rhamnolipid, i.e. 15 mg. At a rhamnolipid concentration of 60 mg, the expression of this gene was reduced by approximately 50-fold.

## Discussion

Various reports have shown that MRSA is one of the most important pathogenic bacteria involved in nosocomial infections. This bacterium is one of the bacteria that can form biofilms. It has been well established that the formation of biofilms by bacteria protects them against the immune system and thus increases antibiotic resistance and the spread of infection and pathogenicity^6^. Due to the increasing resistance of this group of pathogenic bacteria to various antibiotics, the isolation and identification of natural compounds with antibacterial and anti-biofilm activities and understanding their functional mechanism at the cellular and molecular levels is of great importance. One of the beneficial metabolites produced mainly by different strains of *P. aeruginosa* is rhamnolipid biosurfactants with surface tension-reducing properties. They have a high advantage over similar chemical compounds to control biological disease, including low toxicity, high degradability, and effectiveness at temperature, pH, and salinity. In this regard, the focus of the present study is, in particular, on the antibacterial and anti-biofilm activities of rhamnolipid biosurfactants extracted from a native strain of *P. aeruginosa* against MRSA bacterium. Silva et al.^27^ showed that rhamnolipid at 25 °C and a concentration of 0.1% was able to reduce the biofilm mass of *S. aureus* (MTCC 3160) by about 35%. It was also shown that the inhibition rate of *S. aureus* biofilm formation by rhamnolipid biosurfactant depends on the type of culture medium of this bacterium. Rhamnolipid at 25 °C effectively removed 86.9% of the biofilms formed in the skim milk medium, whereas the broth nutrient medium was only able to affect and reduce the biofilm by 35%. This means that rhamnolipid was able to inhibit the biofilm formed by *S. aureus* in skim milk more than the biofilm formed by this bacterium in nutrient broth^27^. The main components of the biofilm matrix structure are polysaccharides, proteins, lipids, and nucleic acids^31^. Kim et al. showed that rhamnolipid at a concentration of 300 µg/mL was able to reduce the number of carbohydrates and protein in the biofilm formed by *P.aeruginosa* (31.6% and 79.6%, respectively)^32^. In another study, Bagheri et al. (2013) reported that rhamnolipid at a concentration of 512 µg/mL was able to affect the growth of *Staphylococcus aureus* ATCC 29213^33^. In the present study, after determining the MIC by plate count, it found that the rhamnolipid biosurfactant was able to affect the growth of MRSA bacteria and inhibit it even at a certain concentration. The results of viable plate count showed that rhamnolipid concentrations of 3% and 12% were the minimum inhibitory concentration (MIC), and minimum bactericidal concentration (MBC), respectively. One of the reasons for not measuring OD at 600 nm to determine the MIC was the colored nature of the biosurfactant, which in normal conditions also showed absorption. Therefore, in order to determine the MIC and MBC accurately, the viable plate count method was tested, observing that the purified rhamnolipid can inhibit the growth of MRSA bacteria.

Previous studies have shown that rhamnolipid at a concentration of 1% (w/v) reduced 57.8% adhesion of *L. monocytogenes* and 67.8% adhesion of *S. aureus* to polystyrene^34^. Rhamnolipids at a concentration of 0.25%, removed 58.5% of the biofilm of *S. aureus*, 26.5% of *L. monocytogenes*, 23.0% of *S. enteritidis*, and 24.0% of the mixed culture after 2 h contact.

For this purpose, the bacteria were treated with rhamnolipid concentrations according to MIC concentration, followed by crystal violet staining of biofilms after 48 h. The ELISA results showed that the rhamnolipid treatments of biofilms with concentrations of 3%, 6%, 12%, and 24%, compared with the biofilm formed by bacteria in the control state, decreased by 10%, 43%, 50%, and 75%, respectively.

Cell viability and membrane permeability were also investigated using flow cytometry using two fluorescent dyes, FDA and PI. In this regard, MIC concentrations of rhamnolipid were assayed. The more the cell membrane loses its integrity after treatment with rhamnolipid, the more PI dye penetrates the cell, because this dye, due to its size, cannot pass through intact membranes. To evaluate the viability of rhamnolipid-treated cells, FDA can cross the membrane of living and dead cells, and fluorescein dye is released and emitted due to the esterase enzyme activity. The results showed that at a rhamnolipid concentration of 3%, compared with the control, the cell survival and permeability were slightly affected. However, increased membrane permeability and decreased cell viability were observed with increasing rhamnolipid concentration. In order to investigate the changes in the expression of quorum sensing genes involved in the formation of bacterial biofilms, MRSA bacteria were treated in the biofilm state with concentrations of rhamnolipid (equivalent to MIC), and for each treatment, a control set was considered. RNA was then extracted and subsequently, cDNA was synthesized. The analysis of the expression profiles of agrC and agrA genes related to agrACDB operon and icaA and icaD genes related to icaABCD operon and srtA gene in biofilm showed a significant reduction. Recent studies have shown that a higher expression of these genes occurs during the infection process of MRSA bacteria as they produce biofilms. Thus, rhamnolipid was able to reduce the expression levels of these genes and inhibit biofilm formation by this bacterium. The agr quorum system is regulated in two ways: (1) non-RNAIII-dependent or agr operon, and (2) RNAIII-dependent^35^. In the first pathway, when the agrA gene is activated, agrABCD operon is triggered, and phenol-soluble modulins (PSMs) are produced by *Staphylococcus* strains. Phenol-soluble modulins (PSMs) are involved in the biofilm formation and pathogenicity of *S. aureus*. In another study, the effect of biosurfactant extracted from *Lactobacillus plantarum* 27167, at a concentration of 10%, was able to reduce the expression of agrA and icaA genes, thus preventing the formation of biofilms in *S. aureus*. However, in this study, as only two genes from the ica and agr pathways were examined, to gain a better understanding of the underlying process, it was necessary to study more genes from both pathways^35,36^. Compared to studies conducted with rhamnolipid extracted from *Pseudomonas* at a concentration of 1.5%, it was able to reduce the expression of these genes. Our results showed that the treatment of bacterial cells with rhamnolipid led to a reduced expression of agrA, and agrC genes so that no biofilm is formed and bacterial pathogenicity would be reduced (Fig. 7).

**Figure 8:**
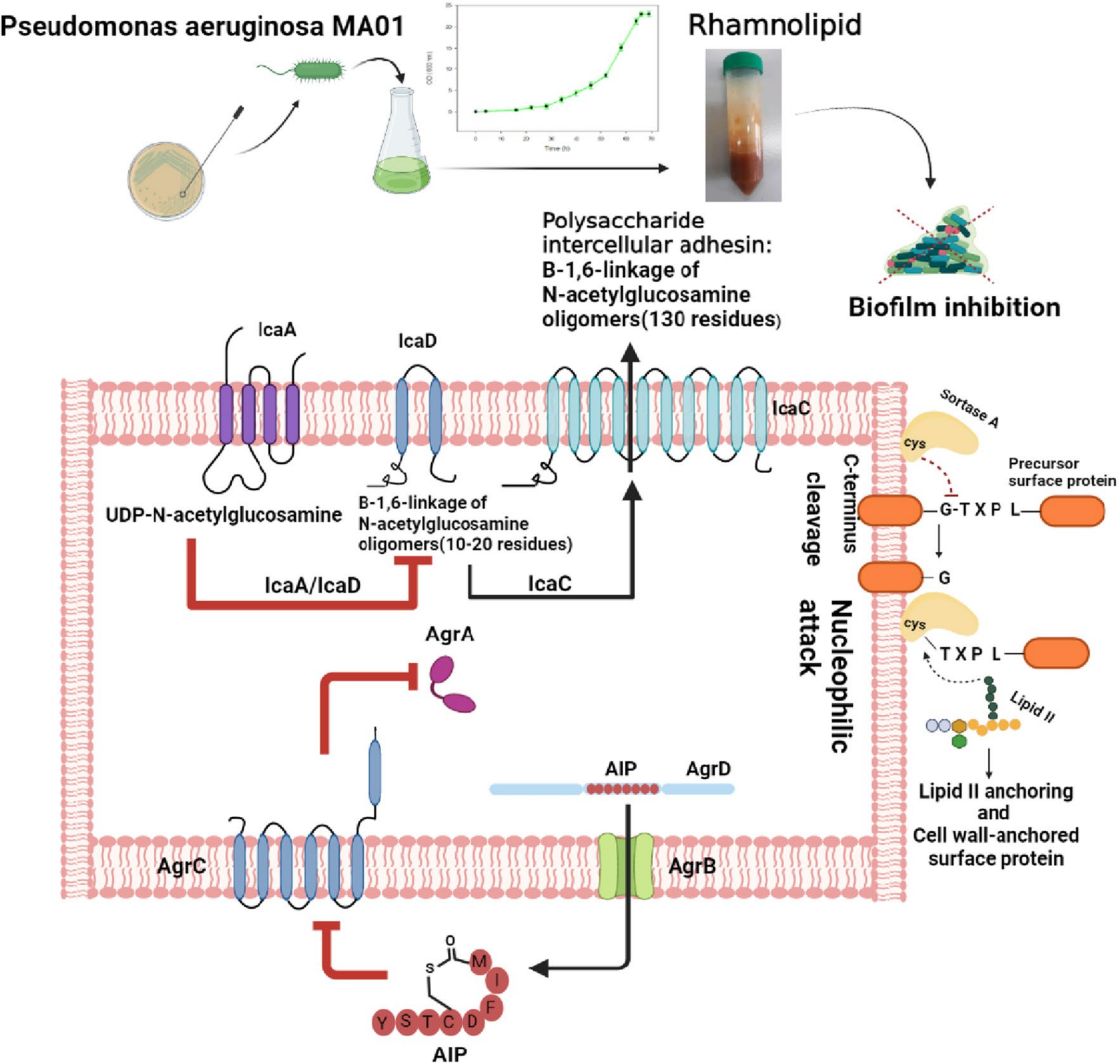
A summary of the research performed. As shown, the extracted rhamnolipid reduces the expression level of the desired genes, icaA and icaD, agrA and agrC, and srtA, thus inhibiting the formation of *S. aureus* biofilms.

The ica (intercellular adhesion operon) operon plays a key role in the formation of biofilms by MRSA. This system is normally active when the bacterium is stressed, triggering biofilm formation. Recent studies have shown that the *ica*A gene is responsible for encoding the N-acetyl glucose aminyl transferase enzyme, which can synthesize N-acetyl glucosamine oligomers from UDP-N- acetyl glucosamine. N-Acetyl glucosamine is an intercellular adhesive polysaccharide that plays a fundamental role in the biofilm formation by MRSA. *Ica*D, which is one of the genes in this operon, cooperates extensively with icaA and intensifies its action^37–40^. In our study, the expression of *ica*A and *ica*D genes was decreased when the bacterium was treated with rhamnolipid as confirmed by real-time PCR data (Fig. 7). The effect of rhamnolipid on the expression of the sortase gene showed a reduced expression of this gene (Fig. 7e). It is believed that one of the factors that inhibit the biofilm formation of *S. aureus* by rhamnolipid is the reduced expression of this gene^21^. When the expression of this gene is reduced, other surface proteins such as ClfA and ClfB will not be able to bind to the cell wall with srtA and thus may affect the formation of biofilms in *S. aureus*^41^. Overall, it can be concluded that the treatment of MRSA bacteria with rhamnolipid at a concentration of 3% (30 mg/mL) would result in reduced expression of the *ica*A gene, so that it is no longer able to encode the N-Acetyl glucose aminyl transferase enzyme, consequently inhibiting the biofilm formation by *S. aureus* (Fig. 8). Because the biofilm formed by the MRSA bacterium causes antibiotic resistance and the spread of pathogenesis, its inhibition by rhamnolipid is of great importance.

## Conclusion

In the current study, it was found that rhamnolipid can affect the expression of genes involved in the formation of MRSA bacterial biofilm, including icaA, icaD, agrA, agrC, and srtA, preventing biofilm formation. In addition, rhamnolipid was shown to be able to penetrate the cell. Given that little research has been done on the exact effect of rhamnolipid, extracted from *Pseudomonas aeruginosa*, on this nosocomial pathogen, the present work could improve our knowledge concerning the inhibition of methicillin-resistant *Staphylococcus aureus*. However, in order to use this bioactive substance to inhibit MRSA bacteria as a therapeutic agent, more studies are needed in the future. Further studies are underway in our laboratory to use the rhamnolipid biosurfactants in much lower concentrations with the combination of some effective antibacterial agents.

## Data Availability

The data sets analyzed during the present study is provided upon request from the corresponding author.

## Abbreviations

MRSA: Methicillin-resistant *Staphylococcus aureus*
PI: Propidium iodide
FDA: Fluorescein diacetate
